# Protecting healing relationships in the age of electronic health records: report from an international conference

**DOI:** 10.1093/jamiaopen/ooz012

**Published:** 2019-07-05

**Authors:** Elizabeth T Toll, Maria A Alkureishi, Wei Wei Lee, Stewart F Babbott, Philip A Bain, John W Beasley, Richard M Frankel, Alice A Loveys, Hedy S Wald, Susan S Woods, William R Hersh

**Affiliations:** 1 Pediatrics and Medicine, The Warren Alpert Medical School of Brown University, Providence, Rhode Island, USA; 2 Pediatrics, The University of Chicago, Chicago, Illinois, USA; 3 Medicine, The University of Chicago, Chicago, Illinois, USA; 4 Medicine, University of Virginia, Charlottesville, Virginia, USA; 5 Internal Medicine, Bozeman Health, Bozeman, Montana, USA; 6 Department of Family Medicine and Community Health, University of Wisconsin, Madison, Wisconsin, USA; 7 Medicine, Indiana University School of Medicine, Indianapolis, Indiana, USA; 8 Family Medicine, The Warren Alpert Medical School of Brown University, Pawtucket, Rhode Island, USA; 9 Child Neurology and Neurodevelopmental Disabilities, Boston Children’s Hospital, Harvard Medical School, Boston, Massachusetts, USA; 10 Medical Informatics, University of New England, Portland, Maine, USA; 11 Department of Medical Informatics and Clinical Epidemiology, Oregon Health and Science University, Portland, Oregon, USA

**Keywords:** patient–practitioner relationship, international experience with electronic health records, burnout, solutions to electronic health record challenges, design, patient–practitioner–computer triad

## Abstract

We present findings of an international conference of diverse participants exploring the influence of electronic health records (EHRs) on the patient–practitioner relationship. Attendees united around a belief in the primacy of this relationship and the importance of undistracted attention. They explored administrative, regulatory, and financial requirements that have guided United States (US) EHR design and challenged patient-care documentation, usability, user satisfaction, interconnectivity, and data sharing. The United States experience was contrasted with those of other nations, many of which have prioritized patient-care documentation rather than billing requirements and experienced high user satisfaction. Conference participants examined educational methods to teach diverse learners effective patient-centered EHR use, including alternative models of care delivery and documentation, and explored novel ways to involve patients as healthcare partners like health-data uploading, chart co-creation, shared practitioner notes, applications, and telehealth. Future best practices must preserve human relationships, while building an effective patient–practitioner (or team)-EHR triad.

## INTRODUCTION AND CONFERENCE DESIGN

The electronic health record (EHR) has profoundly influenced the practice of medicine and patient–practitioner interactions in clinical settings.^1^^,^^2^ Alongside benefits of consolidated patient records, improved communication, and ability to address population health have come unintended consequences, including challenges to the patient–practitioner relationship^3–7^ and, in the United States, a precipitous decline in clinician wellbeing and professional satisfaction.^8–11^ In March 2017, 160 patients, practitioners, educators, technology designers and vendors, government officials, patient advocates, and healthcare stakeholders from the United States and 6 industrialized nations (Canada, United Kingdom, Denmark, Portugal, Israel, and Australia) gathered at The Warren Alpert Medical School of Brown University to explore this pressing topic. Practitioners included physicians, nurses, nurse practitioners, and mental health professionals; medical students, and residents also participated. There were plenary sessions, TED-style talks, symposia, research papers, posters, and digital demonstrations. Conference leaders invited experts, distributed email requests for submissions, and advertised this open conference through mailings, LISTSERVs, social media, and word of mouth. Presenters comprised about half the attendees. International participants were invited because non-US practitioners have had longer and generally more positive EHR experiences than many in the United States. This article summarizes conference conclusions.^12^

## THE VALUE OF THE PATIENT–PRACTITIONER RELATIONSHIP

Patients and practitioners expressed remarkably similar wishes for relationship-based care as technology advances, underscoring the central elements of constructive patient–practitioner interactions.^13^^,^^14^ Both groups want time and space to rekindle or establish trusting relationships, receive or offer eye contact and full attention,^2^^,^^3^^,^^13–17^ and be heard or engage in careful listening.^1^^,^^2^ Patients want to be known as individuals who have valuable firsthand knowledge of their health experiences. Practitioners want to be respected for medical expertise and technical skills but recognized as having human limitations, especially when grappling with healthcare system challenges. Both groups believe clinical encounters should be about patient care and not its documentation.

## EHR BENEFITS TO PATIENTS AND PRACTITIONERS

Patients praised many EHR features, noting that records do not get lost, are accessible from anywhere, and allow patients to participate in their own care by using portals to schedule appointments, review test results, and communicate with practices. Patients imagine contributing further to their EHRs by uploading health materials, creating personalized biographies,^18^ reviewing practitioners’ notes,^19^ and even co-creating notes. Practitioners value EHRs for their legibility, accessibility, consolidated health information, embedded references, decision support, and potential to enhance patient education and understanding.

## EHR CHALLENGES TO THE PATIENT–PRACTITIONER RELATIONSHIP

Many challenges have accompanied the rapid introduction of EHRs into clinical care. Managing the volume of information and juggling the complexities of patient needs and EHR systems, while recording care that justifies reimbursement has overwhelmed US practitioners.^11^^,^^16^^,^^20^^,^^21^ Longer workdays, work after work, more time spent documenting care and completing administrative tasks than face-to-face with patients, and fewer patients seen have left both patients and practitioners feeling that people often play second fiddle to computers.^8–11^^,^^13^^,^^15^^,^^16^^,^^20–27^ Both groups described a distressing decrease in human connections and meaningful interactions.^2^^,^^20–24^ They worry about patient privacy and confidentiality.^4–6^^,^^13^^,^^28^^,^^29^ These trends have contributed significantly to the EHR’s implication in US practitioner burnout.^8–11^^,^^21^^,^^23^^,^^26^^,^^27^^,^^30^

### Roll-out challenges

Conference participants noted that the EHR was introduced into traditional ways of conducting care before rethinking roles, tasks, workflow, workloads, and redundancy. They observed that change is difficult. Implementing untested systems and integrating new tools before having confidence in them is stressful, particularly when patient health is at stake. Furthermore, new systems reveal hidden flaws, for example, poorly controlled patients lost to care. They also present unforeseen challenges like balancing improved access to patient records with maintaining confidentiality^4–6^^,^^28^^,^^29^ or leveraging tools such as templates and copy and paste while creating useful and ethical documentation.^4^^,^^13^^,^^14^

### Design and vendor challenges

The US designers and vendors of small and large EHRs described challenges of designing effective EHRs and interconnected systems. The absence of national standards in areas like preventive measures and laboratory results present additional hurdles.^4^^,^^31^^,^^32^ Regulations imposed by institutions fearing data breaches and subsequent penalties further challenge interoperability and data access,^32^^,^^33^ as has the open market of the past decade, which led to the emergence of hundreds of companies creating a variety of propriety and open source products.^31^^,^^34^ The financial burden of designing software to meet changing guidelines such as meaningful use has hampered competition by smaller, innovative companies. Ultimately, however, fewer dominant EHRs may facilitate interoperability.

### Government challenges

Government presenters described accountability to tax payers and legislators, and the herculean task of developing programs to move health information technology ahead in US regions with vastly different population densities, demographics, geographies, and technological maturity. They described listening to patients’ and practitioners’ needs when devising statewide initiatives such as Colorado’s telehealth bill and Vermont’s improved broadband access initiative, as well as current federal legislative mandates like accountable care, interconnectivity, and data accessibility.

## OBSERVATIONS FROM OTHER NATIONS

International attendees recounted stories from their national and personal EHR experiences. While anecdotal, their reports offered valuable opportunities for sharing insights and collaborative reflection. An Australian participant described the pain of rolling out health information technology infrastructure and adopting EHRs in his nation, but noted that patients welcomed EHRs, believing it would be difficult to practice modern medicine without a computer. A Danish presenter elucidated his government’s well-regarded EHR used in all facilities of their single-payer health system, allowing clinicians easy access to patient records and clinical data to feed directly into national population and public health research and policy development.^35^ A participant from Portugal described that nation’s creation of a similar comprehensive infrastructure, but its progression to a more siloed and less streamlined system because of inconsistent internet access and the establishment of parallel infrastructures for e-prescribing, death records, and epidemiology.

In the United Kingdom, EHRs initially designed by family physicians for patient care have been deployed on a national scale and are well liked and trusted. Anecdotally, as US EHRs have been imported for inpatient use, UK hospitals face some of the same challenges to efficiency and overdocumentation as US users of the same products.

In Israel, EHRs also designed around patient care require many fewer clicks for prescribing, writing orders, and documenting than their US counterparts. Billing is separated from the EHR. Israel’s 12 major health systems, including the Israeli Defense Forces, have successfully interconnected their EHRs, with data based in each home institution but accessible within seconds from any other system.^36^ Thus far there have been no breaches.

A Canadian speaker described innovative efforts to help patients with complex medical problems set visit agendas directly into the EHR.^37^ A UK presenter described a pioneering program in Bangladesh connecting patients to primary and specialty care when signing up for banking.

Perhaps the most striking feature noted by international presenters was that their countries’ EHRs were designed to record patient care; interestingly, clinical notes are, on average, 4 times shorter than US notes.^27^

## SUGGESTED SOLUTIONS

While identifying current challenges to the patient–practitioner relationship, conference attendees expressed the need to move toward a constructive triad of patient–practitioner–computer, or patient-healthcare team-computer in coming years.^38^^,^^39^ Diverse solutions were explored, encouraging all to imagine others.

### Education

Education was cited as a critical component of all solutions. Complex EHR skills require time and effort to master and must be balanced with other medical and systems knowledge clinicians need to learn for their daily work. Institutions must commit time and resources to robust initial and ongoing education of all medical professionals, whether at the outset of training or employment or in mid-career, taking into consideration diverse backgrounds, technological skills, and trends toward inter-professional team care. While software subtleties may allow for more efficiency, users need training and practice in these areas, too, including effective use of embedded EHR feedback tools.^40^

**Figure 1.**
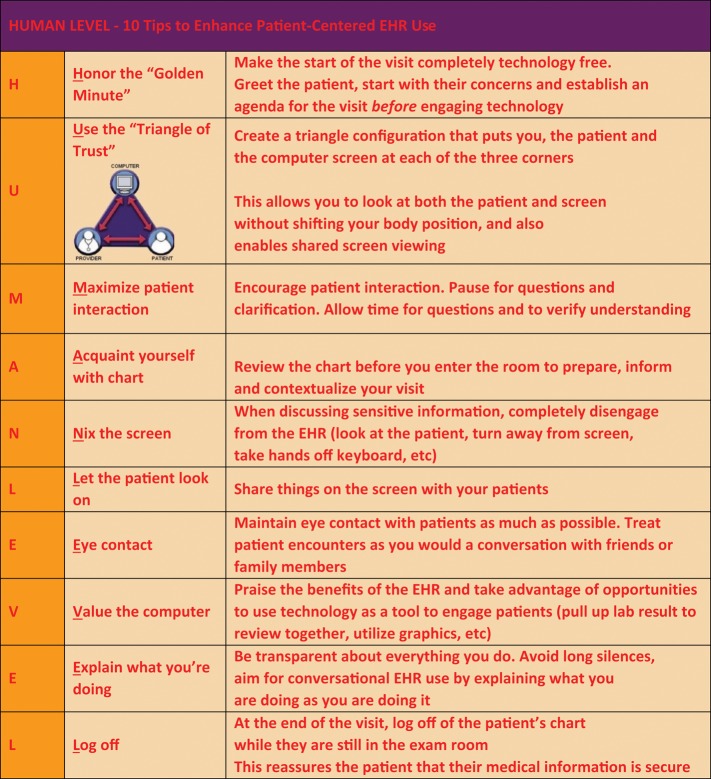
HUMAN LEVEL mnemonic for patient-centered electronic medical record use. *Abbreviation:* EHR: electronic health record. From Mann and Slaboch^41^ and Lee *et al*.^42^

Most importantly, education should focus on effective EHR use during encounters to strengthen rather than detract from patient–practitioner communication.^1^^,^^2^ Tips to create patient-centered EHR best practices are shown in Figure 1.^41^^,^^42^ Optimal use requires personalization, observed evaluation, ongoing feedback, and organizational support. Curricula are being developed at a variety of institutions^43^ (Table 1). Learners need training to create EHR documentation that encourages and conveys their thinking, while selectively employing time-saving tools like voice-to-text, text-forward, and templates.^13^^,^^53^

**Table 1.** Examples of EHR curricula

Institution	Components	Materials and methods
Alpert Medical School of Brown University (Wald et al)^44^	MS3—lecture, behavior grid, OSCE, and narrative/reflective exercise MS4—advanced EHR module with expanded behavior grid, and OSCE	Behavior grid Reflective reading and writing Feedback from multidisciplinary faculty and standardized patient during practice
Hebrew University of Jerusalem (Reis)	Classroom instruction, computer simulation, OSCE, addressing, and using social media Family Medicine resident curriculum expanded with the Doctor in the Digital Age	e-SEGUE tool to assess communication skills, including via EHR^45^^,^^46^
Oregon Health & Science University (Hersh et al)	Curriculum based on defined competencies^47^ implemented in longitudinal curriculum in clinical informatics; begins with access to EHR on first day of medical school^48^ Promotes optimal EHR use throughout medical school using case-based curriculum via the EHR Curricular activities, including EHR-based simulations, teaching skills of medication reconciliation, order entry, chart maintenance, and evidence-based chronic disease management	Interactive lectures Small group workshops Clinical informatics pearls—short use asynchronous online lectures Cases EHR simulation OSCEs
University of Chicago (Alkureishi and Lee)	MS2—lecture, group OSCE^42^^,^^49^ MS3—lecture, capstone individual OSCE^42^^,^^49^ Orientation lecture for all new interns, residents, fellows on best practices and professionalism in documentation integrated into EHR onboarding^50^ PGY1-3 pediatrics—lecture PGY1 Internal Medicine—ambulatory bootcamp lecture Attendings—CME lecture and group OSCE	Interactive lecture on patient-centered EHR use including self-reflection, video examples of poor and ideal behaviors, and discussion OSCE with standardized patients and feedback using validated e-CEX tool^51^
University of Toronto (Shachak et al)	Instruction in communication and computer communication skills	e-SEGUE tool^46^

*Abbreviations:* EHR: electronic health record; MS2: second year medical students; MS3: third year medical students; OSCE: objective structured clinical examination; PGY1: postgraduate Year 1 (interns); PGY2: postgraduate Year 2 residents; PGY3: postgraduate Year 3 residents; e-CEX: Electronic-clinical evaluation exercise; CME: Continuing Medical Education.

### Rethinking documentation

It is time to reconsider the components of a well-crafted note as past information is accessible in the EHR. Conference attendees spoke about filtering information to meet clinician needs; tailoring the style of notes to fit their clinical purposes such as preventive, chronic care, consult, or community based care; and choosing the form that best conveys information. The imperative to separate billing from extent of documentation was noted throughout the meeting.^53^

Until ordering and documenting become more streamlined in US EHRs, it is not realistic—for patients who need full attention or clinicians who are more likely to make mistakes when multi-tasking^3^^,^^20^^,^^53^^,^^54^—to expect clinicians to document fully while attending to patients or for hours at night.^8–11^ Rather, trained scribes or team members co-managing the visit should be documenting.^55^^,^^56^ This may change when EHR charting becomes simpler and features like dictation, virtual assistants, and natural language processing improve.^57^

### EHR design

Attendees stressed the need for clinicians and designers to share ongoing front-line experiences and communication.^20^ For example, streamlined software will allow nurses to enter data in real time rather than copying handwritten logs, and smaller devices can facilitate bedside nursing care while entering data.^58^^,^^59^

### Rethinking job descriptions and workflow

The EHR affords us the opportunity to re-evaluate our work and who does it, improve efficiency, reduce redundancy, and commit to restoring and protecting human interactions between patients and practitioners and among medical professionals. All are integral to excellent patient care and professional satisfaction. Suggested EHR design improvements to support optimal patient care are shown in Table 2.

**Table 2.** Design improvements in EHRs and clinical workflows

	Specific suggestions and examples
EHR improvement	
Streamline EHR log-in	Use fingerprint or tap-and-go cards
Improve usability	Simplify screen graphics Reduce clicks and steps to complete tasks Move non-clinical functions behind the scenes to enhance rapport building, visit flow, documentation, and clinical focus
Tailor information presentation	Present timeline with filterable displays of symptoms, labs, treatments, and responses Embed visualization of data using pictures, graphs, tables, or formats most helpful to user^53^^,^^58^
Match data entry modality with task	Include typing, voice-to-text, and dictating Use artificial intelligence to extract text via natural language processing, select information from a list by mouse-click or touch screen, or record a photo, video, or sketch^53^^,^^57^
Tailor note design to encounter type	Straightforward acute-problem visits (eg, urinary tract infection, pharyngitis), template pre-populated by patient Complex visits note design (eg, diagnostic dilemma, multiple chronic problems): combine unique patient narrative, standard text blocks for routine actions (eg, calculating cardiovascular or surgical risk with display for patient teaching), facilitate history and data synthesis, enhance clinical decision-making (including graphic display), and a brief summation of key findings
Use virtual assistants	To search, retrieve, and apply information like retrieving a previous test, creating referrals (that also forward prescriptions and print out preprocedure instructions), calculating risk profile automatically using chart data, or presenting decision-support tools^57^^,^^58^
Use EHR to educate	Use patient’s results and images to explain symptoms Share internet materials, smart phone applications, Bluetooth technology, photography and videography for communication, shared decision-making tools, and data analysis Cocreate office notes
Protect patient confidentiality	Create confidential tab organized as medical history that can be addended over time, accessible only to patient and practitioners (cannot be forwarded)^53^
Embrace patient contribution^67^ (eg, screening, visit agenda^37^, updated personal data and biographies^18^)	Enable patients to become data providers Offer robust patient portals for secure messaging, requesting prescription refills, appointment scheduling, accessing test results and clinical notes, and reviewing medications, problem lists, and care plans for completeness and accuracy Allow bidirectional data sharing remotely and at point of care (eg, screening, visit agenda, updated personal data and biographies)
Facilitate meaningful documentation	Meld different styles of recording—prose, checklists, templates—that encourage modification to unique situation^53^
Improve interoperability and data sharing	Advance standardization of data presentation^31^ Discourage data blocking^32^^,^^33^ Consider a single national EHR
Workflow improvement	
Previsit preparation	Staff oversees: Completion of labs and tests Requesting patients enter biographical information and complete appropriate screening (eg, mental health, health disparities), and start visit agenda Identify care gaps (immunizations, age-based screening tests, follow-up for previous problems) Team huddles
Task reallocation (“Lean” concept)^a^	Recognize physician, nurse, and staff time and attention as precious and costly resources Reallocate tasks to match professional training, decrease physician administrative burden, and diminish distraction
Additional staff	Enter, review, and manage EHR data, complete order entry, and communicate with patients between visits; can include scribes^55^^,^^60^ or clinical team coordinators^61^^,^^62^ Some models describe 2–3 coprofessionals (eg, medical assistants, nurses, pharmacists) per physician^61^^,^^62^
Reduce administrative burden^11^^,^^30^	Dedicate time during normal hours to address administrative tasks
Facilitate team communication^61^^,^^62^	Create office design to dovetail with workflow, maximize visualization, decrease unnecessary movement, and support teamwork Encourage a variety of face-to-face, telephone, paper, and electronic communication based on efficiency and improved clinical outcomes Colocate team workspaces to enhance verbal communication; reduce ping-ponging computer messages; and promote human contact, clinical consultation, and team connections
Decrease documentation time	Address current regulations linking documentation and billing Optimize scribe and team documentation^55^^,^^60–62^
Maximize EHR placement and usefulness	Enhance communication with large or multiple screen monitors Employ computer as a bridge rather than a barricade to patient information sharing^63^^,^^64^ Use exam-room printers, allowing clinicians to review instructions, teaching materials, and after-visit summary with patient and save time

*Abbreviation:* EHR: electronic health record.

^a^Lean refers to a set of operating philosophies and methods that help create a maximum value for patients by reducing waste and waits.^65^

Lean principles of industrial engineering can help design workplaces to maximize teamwork,^65^ streamline patients’ movement through care, and utilize EHRs to support and educate.^42–51^ As shown in Table 2, the healthcare team works as a unit according to lean principles with tasks allocated at the level of training; all help document. Many clinical settings still rely on paper for reviewing past care, completing forms, and faxing information. When EHR functions, graphics, and information display become more user-friendly, this work can be done once using the computer.^66^

Reimagined care delivery includes teams of medical professionals maximizing their training and skills to support patients and one another.^56^^,^^61^^,^^62^

### The patient as team member

Patients are indispensable team members who can serve as valuable data providers.^67^ This can be achieved by expanding portal functions to enable patients to initiate visit documentation from home or on devices at the point of care. Patients can update demographic, insurance, and biographical information,^18^ or review EHR data and set visit agendas.^37^^,^^53^ The OpenNotes project has pioneered promoting patient access to their clinical notes, including behavioral health notes^68^^,^^69^; remaining challenges include maintaining adolescent and parent confidentiality^6^^,^^29^, and practitioner perceptions that sharing notes impacts full discussions of clinical thinking, differential diagnoses, and psychosocial issues.^68^^,^^70^

Interestingly, patients and practitioners have expressed overall satisfaction with shared notes, finding them respectful and communicative, and patients are better able to adhere to recommendations when they review the thinking behind them.^19^^,^^69^ Patients also identify and correct errors in their records.^19^^,^^69^ Patients have begun sharing photos and videos with clinicians on their smart phones; these can be uploaded into their records—first steps toward having patients co-create documentation. One can imagine a future in which a personal health record belongs to the patient, who shares data with practitioners and institutions, generating care centered around shared decisions, clear communication, and consideration of the patient’s life outside the medical system.

### New technologies

Many ideas were shared among participants across disciplines regarding new technologies. For example, SMS texting is a powerful tool to remind patients about appointments, support behavior modification,^71^ and communicate information. So too, applications (“Apps”) can help patients manage health issues like dietary change, asthma,^72^ diabetes,^73^ menstrual cycles, and anxiety. Some are employed by patients on their own, while others transmit data to practitioners for review. Patients and practitioners need guidance in sorting through the many available products to be confident that apps advance patient health and support clinical care with manageable data loads. Telehealth also holds great promise to connect patients, practitioners, and family members when geographical, medical, and psychological barriers prevent in-person visits.^74^

## SUMMARY

Participants at this groundbreaking conference valued hearing the aspirations, challenges, and perspectives of a diverse group of attendees. They remained committed to protecting the patient–practitioner relationship as the foundation of excellent care and patient and practitioner satisfaction in this age of advancing health information technology. International colleagues demonstrated the value of EHRs with documentation centered around clinical care, seamless interoperability, and prompt data access for patient care and population health. The US attendees were challenged to find ways to decrease clicks, screen shifts, and excessive documentation and improve interconnectivity and data sharing. Conference attendees highlighted the importance of ongoing communication and advocacy by all to ensure a future medical system that preserves the healing power of human relationships, while harnessing the tremendous potential of health information technology to improve medical care and health.

Two commercial products are mentioned in the references.

1. The conference had no connection or commercial relationship with Imagining a Medical Record of the Future (Ref.^57^).
2. Gregory Makoul, PhD, Founder and CEO of PatientWisdom was a conference presenter and received support for travel and housing from The Physicians Foundation, a conference funder, but no additional remuneration (Ref.^18^).
3. The LEVEL mnemonic/behaviors in Figure 1. were originally published as Mann WR, Slaboch J. Computers in the exam room—friend or foe? *Perm J*. 2004; 8 (4): 49–51. Copyright 2004, The Permanente Federation, LLC and is used with permission.
4. The HUMAN LEVEL figure was originally published as Alkureishi M, Lee W, Farnan J, Arora V. Breaking away from the iPatient to care for the real patient: implementing a patient-centered EMR use curriculum. *MedEdPORTAL* 2014; 10: 995 and is used with permission from Maria Alkureishi, MD and Wei Wei Lee MD/MPH.

## FUNDING

Funding for The Patient, the Practitioner, and the Computer: Holding on to the Core of Our Healing Professions in a Time of Technological Change (March 17–19, 2017) was received from: The Warren Alpert Medical School of Brown University (Office of the Associate Dean for Medical Education, Department of Family Medicine, Department of Pediatrics), American Medical Association, Arnold P. Gold Foundation, Josiah Macy Jr. Foundation, The Physicians Foundation, Rhode Island Foundation, Rhode Island Quality Institute, Rhode Island Medical Imaging, University Emergency Medicine Foundation (now Brown Emergency Medicine), University Medicine Foundation (now Brown Medicine), University Orthopedics, Andrew and Shelley Sigal, and an Anonymous Donor.

*Conflict of interest statement*. E.T.T. received salary support from the Rhode Island Foundation and salary support and housing costs from The Physicians Foundation. Both organizations were conference funders. She is a member of The Warren Alpert Medical School Departments of Pediatrics and Medicine, both conference funders. M.A.A. received travel and housing support from The Physicians Foundation and received grant support from the Arnold P. Gold Foundation, both conference funders. W.W.L. received travel and housing support from The Physicians Foundation and received grant support from the Arnold P. Gold Foundation, both conference funders. S.F.B. received travel and housing support from The Physicians Foundation, a conference funder. P.A.B. received travel and housing support from The Physicians Foundation, a conference funder. J.W.B. received travel and housing support from The Physicians Foundation, a conference funder. R.M.F. and S.S.W. have no conflict of interest to declare. A.A.L. received travel and housing support from The Physicians Foundation, a conference funder. H.S.W. received housing support from The Physicians Foundation, a conference funder. W.R.H. received travel and housing support from The Physicians Foundation, a conference funder.
